# Sex-specific differences and long-term outcome of patients with coronary artery disease and chronic kidney disease: the Coronary Artery Disease and Renal Failure (CAD-REF) Registry

**DOI:** 10.1007/s00392-021-01864-5

**Published:** 2021-05-26

**Authors:** Christiane Engelbertz, Hans O. Pinnschmidt, Eva Freisinger, Holger Reinecke, Boris Schmitz, Manfred Fobker, Roland E. Schmieder, Karl Wegscheider, Günter Breithardt, Hermann Pavenstädt, Eva Brand

**Affiliations:** 1 Department of Cardiology I – Coronary and Peripheral Vascular Disease, Heart Failure, University Hospital Muenster, Cardiol, Muenster, Germany; 2 Department of Medical Biometry and Epidemiology, University Medical Centre Hamburg-Eppendorf, Hamburg, Germany; 3 Institute of Sports Medicine, Molecular Genetics of Cardiovascular Disease, University Hospital Muenster, Muenster, Germany; 4 Center of Laboratory Medicine, University Hospital Muenster, Muenster, Germany; 5 Department of Nephrology and Hypertension, University of Erlangen-Nuernberg, Erlangen, Germany; 6 Department of Nephrology, Hypertension, and Rheumatology, University Hospital Muenster, Muenster, Germany; 7 Allg. Innere Medizin sowie Nieren- und Hochdruckkrankheiten und Rheumatologie, Medizinische Klinik D, Universitätsklinikum Münster, Albert-Schweitzer-Campus 1, Gebäude A1, 48149 Münster, Germany

**Keywords:** Coronary artery disease, Chronic kidney disease, Sex, Long-term mortality, Treatment

## Abstract

**Background:**

Cardiovascular morbidity and mortality are closely linked to chronic kidney disease (CKD). Sex-specific long-term outcome data of patients with coronary artery disease (CAD) and CKD are scarce.

**Methods:**

In the prospective observational multicenter **C**oronary **A**rtery **D**isease and **RE**nal **F﻿**ailure (CAD-REF) Registry, 773 (23.1%) women and 2,579 (76.9%) men with angiographically documented CAD and different stages of CKD were consecutively enrolled and followed for up to 8 years. Long-term outcome was evaluated using survival analysis and multivariable Cox-regression models.

**Results:**

At enrollment, women were significantly older than men, and suffered from more comorbidities like CKD, hypertension, diabetes mellitus, and multivessel coronary disease. Regarding long-term mortality, no sex-specific differences were observed (Kaplan–Meier survival estimates: 69% in women vs. 69% in men, *p*_log-rank_ = 0.7). Survival rates decreased from 89% for patients without CKD at enrollment to 72% for patients with CKD stages 1–2 at enrollment and 49% for patients with CKD stages 3–5 at enrollment (*p*_log-rank_ < 0.001). Cox-regression analysis revealed that sex or multivessel coronary disease were no independent predictors of long-term mortality, while age, CKD stages 3–5, albumin/creatinine ratio, diabetes, valvular heart disease, peripheral artery disease, and left-ventricular ejection fraction were predictors of long-term mortality.

**Conclusions:**

Sex differences in CAD patients mainly exist in the cardiovascular risk profile and the extent of CAD. Long-term mortality was not depended on sex or multivessel disease. More attention should be given to treatment of comorbidities such as CKD and peripheral artery disease being independent predictors of death.

*Clinical Trail Registration* ClinicalTrials.gov Identifier: NCT00679419

**Graphic abstract:**

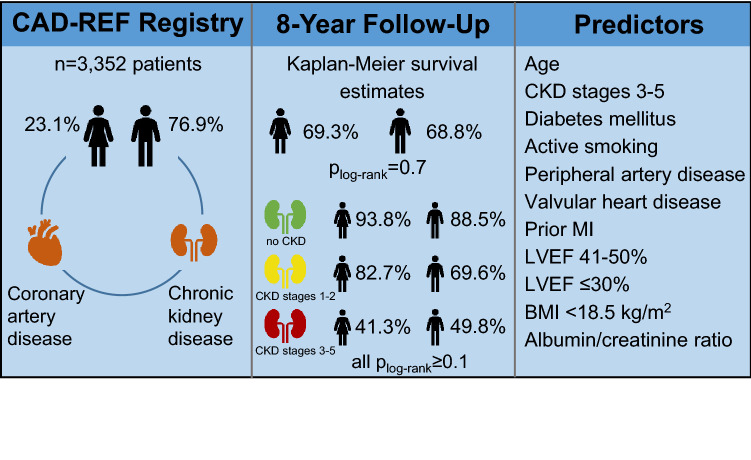

**Supplementary Information:**

The online version contains supplementary material available at 10.1007/s00392-021-01864-5.

## Introduction

Coronary artery disease (CAD) and chronic kidney disease (CKD) are frequently associated. The prevalence of CAD as well as of CKD differs in men and women. Women have a higher prevalence of CKD than men [1], whereas the prevalence of CAD is higher in men than in women [2]. Both morbidities share some risk factors, e.g., higher age, male sex, smoking, hypertension, and diabetes mellitus [2, 3], and patients with CKD are more likely to die from cardiovascular disease than to progress to end-stage renal disease [4]. The long-term outcome of women and men with CAD is still a matter of debate: some studies and registries reported higher mortality for women with CAD, others reported no difference [5–8]. Data on patients suffering from both, CAD and CKD, are rarely presented, because patients with CKD are often excluded from studies. Therefore, the influence of renal function on long-term outcome of women and men with CAD is not well studied and needs clarification.

In 2008, the prospective, observational multicenter German **C**oronary **A**rtery **D**isease and **RE**nal **F**ailure (CAD-REF) Registry was established to evaluate the impact of CKD on the manifestation, progression, and general outcome of patients with CAD [9]. Patients with angiographically documented CAD ≥ 50% stenosis in at least one coronary artery were registered, classified by their estimated glomerular filtration rate (eGFR) and followed for up to 96 months. The main objective of this report was to analyze sex-specific differences in baseline characteristics, medical treatment, and long-term mortality of CAD patients with varying degrees of renal disease.

## Materials and methods

The German CAD-REF Registry (ClinicalTrials.gov identifier number NCT00679419, http://clinicaltrials.gov), a multicenter, prospective, observational registry, included patients with an angiographically documented ≥ 50% stenosis in at least one coronary artery. The patients were classified according to their eGFR and proteinuria into either a control group with normal renal function or one of two CKD categories (CKD stages 1–2 or CKD stages 3–5, determination see below). A detailed description of the trial design [9] and baseline characteristics [10] has been published elsewhere. In brief, 3,352 patients of European/white descent aged ≥ 18 years were enrolled at 32 cardiological recruiting centers distributed all over Germany. All patients gave written informed consent prior to their inclusion. Urine, serum, and EDTA-blood samples of each patient were collected prior to coronary angiography. Patients with organ transplantations other than kidney transplantation, with immunosuppressive therapy apart from immunosuppressive therapy after kidney transplantation, with polycystic renal disease, with known malignant tumors as well as pregnant or breastfeeding patients were excluded from the registry.

### Data collection

Before coronary angiography, demographic characteristics, anthropometric data, cardiovascular risk factors, medical history, standard laboratory parameters of serum and urine samples, and medication were recorded. Data on the degree and localization of stenoses were collected from coronary angiograms according to the Cardiology Audit and Registration Data Standards (CARDS) [11]. At hospital discharge, data on medication were recorded. Follow-up data were collected by questionnaire and telephone calls.

Data collection was performed by the IKKF Institute GmbH, Munich, Germany, and the University Hospital Muenster, Muenster, Germany. Primary route of data entry was done through a web-based interface.

### Renal function and proteinuria

Serum creatinine was used to estimate the GFR according to the Chronic Kidney Disease Epidemiology Collaboration (CKD-EPI) formula [12, 13].

Proteinuria was determined using a dipstick test. Proteinuria could not be determined in ten patients because of no residual urine.

For analysis, patients were classified into three categories of CKD: patients without CKD had eGFR ≥ 90 ml/min/1.73 m^2^ and no proteinuria, patients with CKD stages 1–2 had eGFR ≥ 90 ml/min/1.73 m^2^ and proteinuria or eGFR 60–89 ml/min/1.73 m^2^, and patients with CKD stages 3–5 had eGFR < 60 ml/min/1.73 m^2^ or were on dialysis.

### Definition of cardiovascular risk factors

The cardiovascular risk factors were documented based on questionnaires and patients’ records. Definitions of the cardiovascular risk factors are found in the Supplemental Material.

### Data and statistical analyses

All statistical analyses were done using SPSS version 25 and 27 (IBM Corporation, Armonk, NY, USA). Right-skewed continuous variables (creatinine, albumin/creatinine ratio, and protein/creatinine ratio) were log10-transformed prior to further analyses. Baseline characteristics of patients were described by presenting means and 95% confidence intervals of continuous variables, after back-transformation if applicable, as well as absolute and percentage frequency distributions of categorical variables. For continuous dependent variables, comparisons of sex and CKD stages within sex were done based on F tests and associated p values, using the procedure UNIANOVA. For dichotomous dependent variables, comparisons were made based on logistic regression analyses [14], using procedure LOGISTIC REGRESSION. For ordinal dependent variables, procedure GENLIN was used with multinomial distribution and cumlogit link. For nominal variables, procedure NOMREG was used. The p values associated with the respective analyses are reported. These analyses were unadjusted as well as adjusted for age where applicable. Survival of female and male patients was analyzed for the whole cohort and by CKD category (no CKD, CKD stages 1–2, CKD stages 3–5) using the Kaplan–Meier method, comparing sexes by log-rank tests [15]. Survival was further analyzed by multivariable Cox-regression analysis, forcing the independent variables sex, CKD, and all potential confounders into the regression equation and also testing the interactions between sex and all other variables. However, none of the interactions was found to be statistically significant. Missing values occurred when a patient failed to answer a question or when a laboratory value was not obtained. While univariable analyses such as those comparing baseline variables by sex were based on the available non-missing data, multivariable Cox-regression analyses were done on five multiply imputed data sets (imputed by the fully conditional specification method). Hazard ratios with 95% confidence intervals and p values of the pooled results are reported. A two-sided *p* ≤ 0.05 was considered statistically significant.

## Results

### Baseline characteristics

Between January 2008 and May 2011, 773 (23.1%) women and 2,579 (76.9%) men with a ≥ 50% stenosis in at least one coronary artery were consecutively enrolled without preselection (Table 1). Compared to men, women were significantly older at time of enrollment (69.9 vs. 66.3 years, *p* < 0.001), had a lower eGFR (65.7 vs. 73.3 ml/min/1.73 m^2^, *p* < 0.001) and more often presented with severe CKD (39.1% vs. 26.9%), had a higher prevalence of hypertension (87.5% vs. 82.2%, *p* = 0.001), diabetes mellitus (28.8% vs. 24.6%, *p* = 0.02), and valvular heart disease (16.2% vs. 12.8%, *p* = 0.02), and a lower prevalence of prior myocardial infarction (26.5% vs. 34.3%, *p* < 0.001), previous coronary artery bypass grafting (CABG; 16.3% vs. 21.6%, *p* = 0.001) and percutaneous coronary intervention (PCI; 39.8% vs. 46.0%, *p* = 0.002). Women were significantly less often smokers (32.2% vs. 60.9%, *p* < 0.001), drank less alcohol (43.1% vs. 63.7%, *p* < 0.001), and were more often physically inactive (79.7% vs. 75.2%, *p* = 0.02) compared to men (Table 1).

**Table 1:** Patient characteristics and renal laboratory parameters at the time of enrollment

	Overall population	Women	Men	*p* value
**Baseline parameters**				
Patients, *n*(% of all)	3,352 (100.0)	773 (23.1)	2,579 (76.9)	
Age, mean (95% CI), years *n* (women) 773, *n* (men) 2,579	67.1 (66.8–67.5)	69.9 (69.1–70.6)	66.3 (65.9–66.7)	** <0.001**
Age ≤50 years, *n* (%)	258 (7.7)	42 (5.4)	216 (8.4)	**0.008**
BMI, kg/m^2^; *n* (women) 767, *n* (men) 2567				**<0.001**
<18.5 (underweight)	17 (0.5)	3 (0.4)	14 (0.5)	
18.5–24.9 (normal weight)	733 (22.0)	200 (26.1)	533 (20.8)	
25–29.9 (pre-obesity)	1,558 (46.7)	295 (38.5)	1,263 (49.2)	
30–34.9 (obesity class I)	764 (22.9)	184 (24.0)	580 (22.6)	
35–39.9 (obesity class II)	219 (6.6)	69 (9.0)	150 (5.8)	
≥40 (obesity class III)	43 (1.3)	16 (2.1)	27 (1.1)	
WHR, mean (95% CI) *n* (women) 540, *n* (men) 1,772	0.99 (0.98–0.99)	0.94 (0.93–0.95)	1.00 (1.00–1.00)	** <0.001**
Systolic blood pressure, mean (95% CI), mmHg *n* (women) 772, *n* (men) 2,574	134.2 (133.6–134.9)	135.9 (134.4–137.4)	133.7 (133.0–134.5)	**0.009**
Diastolic blood pressure, mean (95% CI), mmHg *n* (women) 772, *n* (men) 2,572	76.6 (76.2–77.0)	76.1 (75.2–76.9)	76.8 (76.3–77.2)	0.1
Pulse pressure, mean (95% CI), mmHg *n* (women) 772, *n* (men) 2,572	57.6 (57.1–58.2)	59.8 (58.6–61.1)	57.0 (56.4–57.6)	** <0.001**
**Cardiovascular risk factors**				
Arterial hypertension, *n* (%)	2,794 (83.4)	676 (87.5)	2,118 (82.2)	**0.001**
Diabetes mellitus, *n* (%)	856 (25.6)	223 (28.8)	633 (24.6)	**0.02**
Hyperlipidemia, *n* (%)	2,178 (67.8)	500 (68.3)	1,678 (67.6)	0.7
Tobacco use (former or active), *n* (%)	1,769 (54.3)	242 (32.2)	1,527 (60.9)	** <0.001**
Alcohol consumption, n (%)	1,611 (59.0)	272 (43.1)	1,339 (63.7)	** <0.001**
Physical inactivity, *n* (%)	2,078 (76.2)	498 (79.7)	1,580 (75.2)	**0.02**
Family history of CAD, *n* (%)	1,176 (42.5)	292 (45.7)	884 (41.6)	0.06
**Cardiovascular events**				
Previous stroke, *n* (%)	188 (5.6)	43 (5.6)	145 (5.6)	0.9
Previous MI, *n* (%)	1,086 (32.5)	205 (26.5)	881 (34.3)	** <0.001**
Previous CABG, *n* (%)	682 (20.3)	126 (16.3)	556 (21.6)	**0.001**
Previous PCI, *n* (%)	1,494 (44.6)	308 (39.8)	1,186 (46.0)	**0.002**
Valvular heart disease, *n* (%)	454 (13.5)	125 (16.2)	329 (12.8)	**0.02**
Previous valve replacement, *n* (%)	48 (1.4)	11 (1.4)	37 (1.4)	0.9
Pacemaker, *n* (%)	232 (6.9)	49 (6.3)	183 (7.1)	0.5
PAD, *n* (%)	350 (10.5)	78 (10.1)	272 (10.6)	0.7
**Renal laboratory parameters**				
Creatinine, mean (95% CI), mg/dl n (women) 773, *n* (men) 2,579	1.1 (1.0–1.1)	0.9 (0.9–1.0)	1.1 (1.1–1.1)	** <0.001**
eGFR, mean (95% CI), ml/min/1.73 m^2^ n (women) 773, *n* (men) 2,579	71.5 (70.8–72.3)	65.7 (64.1–67.3)	73.3 (72.4–74.1)	** <0.001**
CKD				** <0.001**
No CKD, n (%)	629 (18.8)	96 (12.4)	533 (20.7)	
CKD stages 1–2, *n* (%)	1,726 (51.5)	375 (48.5)	1,351 (52.4)	
CKD stages 3–5, *n* (%)	997 (29.7)	302 (39.1)	695 (26.9)	
Proteinuria, *n* (%)	637 (19.1)	151 (19.7)	486 (18.9)	0.6
Albumin/creatinine ratio, mean (95% CI), mg/g *n* (women) 349, *n* (men) 1,199	33.8 (31.5–36.3)	40.5 (35.1–46.7)	32.1 (29.6–34.8)	**0.007**
Protein/creatinine ratio, mean (95% CI), mg/g *n* (women) 721, *n* (men) 2,392	130.4 (126.7–134.3)	169.7 (159.6–180.5)	120.5 (116.7–124.4)	** <0.001**

BMI, body mass index; CABG, coronary artery bypass graft; CAD, coronary artery disease; CI, confidence interval; eGFR, estimated glomerular filtration rate determined by CKD-EPI formula; MI, myocardial infarction; PCI, percutaneous coronary intervention; PAD, peripheral artery disease; WHR, waist-to-hip ratio

Some of the differences in risk factor distribution could be caused by the fact that women were on average 4 years older than men. After age adjustment, the risk factors diabetes mellitus, physical inactivity, and valvular heart disease were no longer significantly different between men and women (Supplementary Table 1).

### Coronary angiography, treatment, and outcome after angiography

Multivessel CAD was found in 67.5% of women and in 76.5% of men (p < 0.001; Table 2). About half of all patients with multivessel CAD had CKD stages 1–2 regardless of sex, whereas CKD stages 3–5 were more prominent in women than in men (40.2% vs. 28.1%). Women had more often normal left-ventricular ejection fraction (> 50%) than men (72.6% vs. 59.9%), whereas men had more than twice as often a severely reduced left-ventricular ejection fraction (≤ 30%) than women (4.2% in women vs. 9.4% in men, *p* < 0.004; Table 2).

**Table 2:** Cardiological data, treatment after/during index angiography, in-hospital complications, and discharge

	Overall population	Women	Men	*p*-value_d_
Patients, *n* (% of all)	3,352 (100.0)	773 (23.1)	2,579 (76.9)	
**Cardiological data**				
Indication for coronary angiography, emergency intervention, *n* (%)	714 (21.3)	167 (21.6)	547 (21.2)	0.8
Multivessel coronary artery disease, *n* (%)	2,494 (74.4)	522 (67.5)	1,972 (76.5)	** <0.001**
No CKD, n (%)	432 (17.3)	55 (10.5)	377 (19.1)	
CKD stages 1–2, n (%)	1,297 (52.0)	257 (49.2)	1,040 (52.7)	
CKD stages 3–5, n (%)	765 (30.7)	210 (40.2)	555 (28.1)	
LVEF, *n* (%)	2,727			**0.004**
Normal (>50%)	1,712 (62.8)	450 (72.6)	1,262 (59.9)	
Slightly reduced (41–50%)	621 (22.8)	106 (17.1)	515 (24.4)	
Moderately reduced (31–40%)	169 (6.2)	38 (6.1)	131 (6.2)	
Severely reduced (≤ 30%)	225 (8.3)	26 (4.2)	199 (9.4)	
**Treatment after/during index angiography**				
PCI performed, *n* (%)	2,281 (68.0)	538 (69.6)	1,743 (67.6)	0.3
Performed stenting, n (%)	1,984 (87.0)	472 (87.7)	1,512 (86.7)	0.9
Bare metal stent, *n* (%)	865 (43.6)	191 (40.5)	674 (44.6)	0.1
Drug eluting stent, *n* (%)	1,178 (59.4)	297 (62.9)	881 (58.3)	0.07
Intervened arteries (LAD, LCX, RCA)				0.5
One, *n* (%)	1,788 (90.1)	423 (89.6)	1,365 (90.3)	
Two, *n* (%)	177 (8.9)	43 (9.1)	134 (8.9)	
Three, *n* (%)	19 (1.0)	6 (1.3)	13 (0.9)	
CABG performed, *n* (%)	344 (10.3)	80 (10.4)	264 (10.2)	0.9
**In-hospital complications**				
PCI after index intervention, *n* (%)	124 (3.7)	23 (3.0)	101 (3.9)	0.2
CABG after index intervention, *n* (%)	98 (2.9)	20 (2.6)	78 (3.0)	0.5
Requiring dialysis after index intervention, *n* (%)	5 (0.1)	1 (0.1)	4 (0.2)	0.9
MI during/after index intervention, *n* (%)	7 (0.2)	2 (0.2)	5 (0.3)	0.7
Stroke after index intervention, *n* (%)	5 (0.1)	2 (0.3)	3 (0.1)	0.4
**Discharge**	3,350	773	2577	0.8
Discharged alive, n (%)	3,037 (90.7)	697 (90.2)	2,340 (90.8)	
In-hospital death, n (%)	8 (0.2)	4 (0.5)	4 (0.2)	
Discharged to another hospital/medical rehabilitation measures, n (%)	305 (9.1)	72 (9.3)	233 (9.0)	

Multivessel coronary artery disease covers two- and three-vessel disease and main stem disease

CABG, coronary artery bypass graft; LAD, left anterior descending artery; LCX, left circumflex artery; LEVF, left-ventricular ejection fraction; PCI, percutaneous coronary intervention; RCA, right coronary artery

Data regarding treatment during and after index angiography revealed no differences between women and men (Table 2). A PCI was performed in 69.6% of all women and 67.6% of all men (*p* = 0.3). A CABG was performed at almost equal rates in both sexes (10.4% vs. 10.2%, *p* = 0.9). With advanced CKD (regarding the categories no CKD, CKD stages 1–2, CKD stages 3–5), patients received less PCIs, but more CABGs (Supplemental Fig. 1a, 1b).

**Fig. 1:**
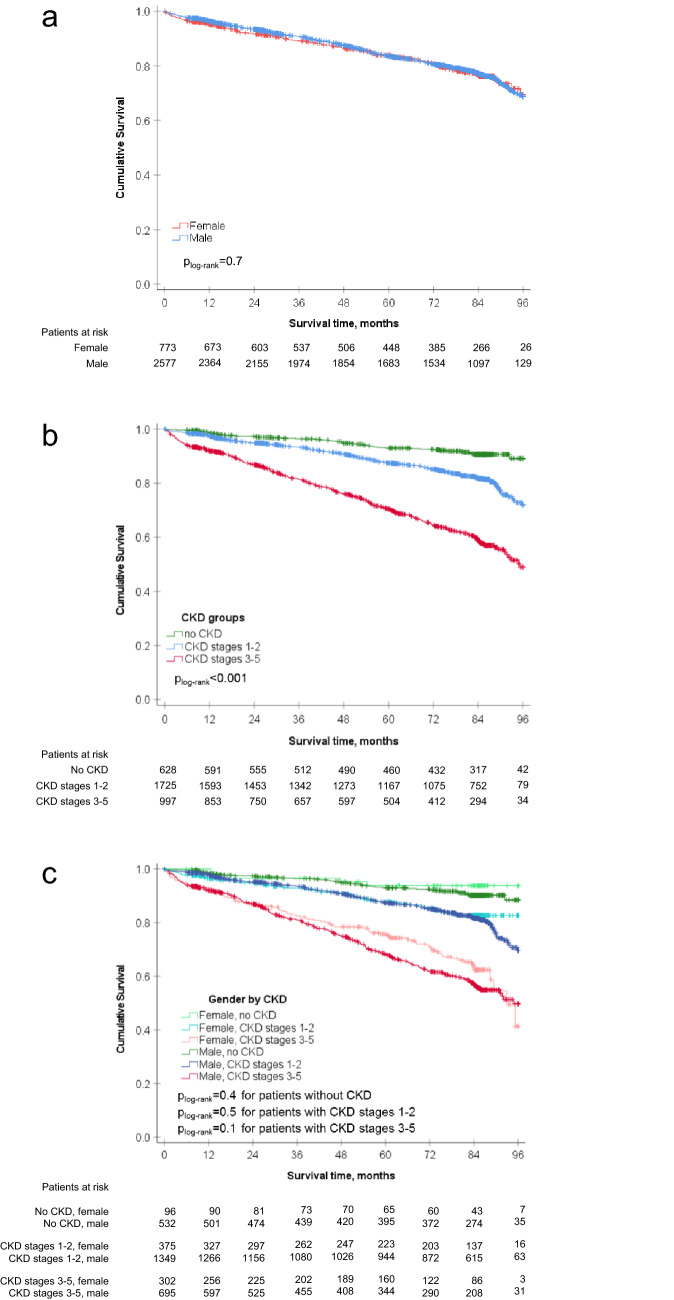
Kaplan–Meier survival analyses. **a** Kaplan–Meier curve for the cumulative survival of women (red line) and men (blue line) during 8-year follow-up. There was no difference in survival between women and men. **b** Kaplan–Meier curve for the cumulative survival of patients according to their renal status at enrollment. Cumulative hazard was significantly different between the three groups. (p_log-rank_ < 0.001). Green line displays patients without CKD, blue line displays patients with CKD stages 1–2, and red line displays patients with CKD stages 3–5. **c** Kaplan–Meier curve for the cumulative survival of women and men according to their renal status at enrollment. There was no difference in the hazards for women and men within the same CKD group. Light green line displays women without CKD, dark green line displays men without CKD, light blue line displays women with CKD stages 1–2, dark blue line displays men with CKD stages 1–2, light red line displays women with CKD stages 3–5, and dark red line displays men with CKD stages 3–5

In-hospital complications and outcome after in-hospital treatment also showed no differences between women and men (Table 2). Stroke or myocardial infarction after index intervention were very low (5 and 7 patients, respectively, Table 2). Only 8 patients died during their in-hospital stay (Table 2).

After age adjustment (Supplementary Table 2), a significant difference between women and men existed also for performed PCI during index angiography (p = 0.04).

### Medication at enrollment and at hospital discharge

Prescription rates of antihypertensive, antithrombotic, antihyperlipidemic, and diuretic drugs were significantly higher after hospital discharge than at enrollment (for all *p* value (visit) < 0.05, except for angiotensin II receptor blocker, Table 3). Regarding antihypertensive drug types, angiotensin-converting enzyme-inhibitors were less often prescribed to women than men (*p* < 0.001), whereas beta-blockers were significantly more often applied in women than men (*p* = 0.03). Diuretics were taken more often by women (*p* = 0.03) and statins were prescribed significantly less often to women than to men (*p* = 0.03).

**Table 3:** Medical treatment at enrollment and at hospital discharge

	Overall population		Women		Men		p value (sex)	p value (visit)
	Enrollment	Discharge	Enrollment	Discharge	Enrollment	Discharge		
Antihypertensive drug, all, *n* (%)	3,013 (89.9)	3,287 (98.1)	697 (90.2)	754 (97.5)	2,316 (89.8)	2,533 (98.2)	0.8	** <0.001**
ACE inhibitor, *n* (%)	1,902 (56.7)	2,299 (68.6)	405 (52.4)	506 (65.5)	1,497 (58.0)	1,793 (69.5)	** <0.001**	** <0.001**
AT1 receptor blocker, *n* (%)	685 (20.4)	742 (22.1)	167 (21.6)	181 (23.4)	518 (20.1)	561 (21.8)	0.2	0.09
Beta-blocker, *n* (%)	2,439 (72.8)	2,884 (86.0)	583 (75.4)	675 (87.3)	1,856 (72.0)	2,209 (85.7)	**0.03**	** <0.001**
Calcium channel blocker, *n* (%)	258 (7.7)	305 (9.1)	69 (8.9)	79 (10.2)	189 (7.3)	226 (8.8)	0.06	**0.04**
Loop diuretic, *n* (%)	625 (18.6)	723 (21.6)	166 (21.5)	196 (25.4)	459 (17.8)	527 (20.4)	** <0.001**	**0.003**
Diuretic, other (thiazides, potassium-sparing), *n* (%)	1,178 (35.1)	1,358 (40.5)	290 (37.5)	332 (42.9)	888 (34.4)	1,026 (39.8)	**0.03**	** <0.001**
Platelet aggregation inhibitor and/or anticoagulant, *n* (%)	2,794 (83.4)	3,237 (96.6)	634 (82.0)	745 (96.4)	2,160 (83.8)	2,492 (96.6)	0.2	** <0.001**
Platelet aggregation inhibitor ASA, *n* (%)	2,569 (76.6)	3,062 (91.3)	585 (75.7)	695 (89.9)	1,984 (76.9)	2,367 (91.8)	0.1	** <0.001**
Platelet aggregation inhibitor clopidogrel, *n* (%)	1,441 (43.0)	2,330 (69.5)	322 (41.7)	548 (70.9)	1,119 (43.4)	1,782 (69.1)	0.9	** <0.001**
Anticoagulant (heparin, vitamin K antagonist and/or NOAC), *n* (%)	548 (16.3)	695 (20.7)	128 (16.6)	169 (21.9)	420 (16.3)	526 (20.4)	0.4	** <0.001**
Statins, *n* (%)	2,282 (68.1)	2,817 (84.0)	512 (66.2)	633 (81.9)	1,770 (68.6)	2,184 (84.7)	**0.03**	** <0.001**

ACE, angiotensin-converting enzyme; ASA, acetylsalicylic acid; AT1, angiotensin II receptor type 1; HMG-CoA, 3-hydroxy-3-methylglutaryl-coenzyme A; NOAC, “non-vitamin K antagonist” oral anticoagulant

Of the three guideline-recommended drug classes for treatment of CAD, only statins with an overall prescription rate of 84.0% were insufficiently prescribed after hospital discharge; more than 95% of all patients were discharged with a prescription for a platelet aggregation inhibitor and/or anticoagulant and an antihypertensive drug (Table 3).

### Long-term overall survival

Follow-up data were available for 3,350 (99.9%) patients. Mean follow-up time was 83.2 months (95% confidence interval 82.3–84.1 months). In total, 663 (19.8%) patients deceased, thereof 144 (18.6%) women and 519 (20.1%) men. Kaplan–Meier estimated 8-year survival rate was 69.3% in women and 68.8% in men (*p*_log-rank_ = 0.7, Fig. 1a). Regarding renal function at enrollment, survival rates decreased from 89.2% for patients without CKD to 71.9% for patients with CKD stages 1–2 and 49.0% for patients with CKD stages 3–5 (*p*_log-rank_ < 0.001, Fig. 1b). There was no difference in survival rates between women and men without CKD (93.8% vs. 88.5%, *p*_log-rank_ = 0.4), with CKD stages 1–2 (82.7% vs. 69.6%, *p*_log-rank_ = 0.5) and with CKD stages 3–5 (41.3% vs. 49.8%, p_log-rank_ = 0.1, Fig. 1c).

Cox-regression analysis of long-term mortality showed no significant difference between sexes (hazard ratio 0.913, 95% confidence interval 0.744–1.120; p = 0.4, Table 4). Increased mortality was associated with higher age, CKD stages 3–5, albumin/creatinine ratio, diabetes mellitus, active smoking, prior MI, valvular heart disease, peripheral artery disease, and slightly as well as severely reduced left-ventricular ejection fraction. Physical activity and a family history of CAD reduced the risk for mortality (Table 4). A power calculation for a two-sided log-rank test on the actual data indicated that a hazard ratio ≤ 0.78 or ≥ 1.26 for female sex (i.e., if the mortality risk of women was ≤ 22% or ≥ 26%, respectively, compared to men) would be detectable within our study with at least 80% power (power = probability of rejecting the null hypothesis when it is false).

**Table 4:** Multivariable Cox-regression analysis of long-term mortality

	Hazard ratio (95% CI)	*p* value
Sex (female)	0.913 (0.744–1.120)	0.4
Age	1.055 (1.043–1.066)	** <0.001**
CKD		** <0.001**
No CKD	1	
CKD stages 1–2	1.180 (0.853–1.634)	0.3
CKD stages 3–5	1.713 (1.209–2.426)	**0.002**
BMI		0.2
18.5–24.9 (normal weight)	1	
<18.5 (underweight)	2.451 (1.063–5.652)	**0.04**
25–29.9 (pre-obesity)	0.895 (0.731–1.097)	0.3
30–34.9 (obesity class I)	0.991 (0.786–1.250)	0.9
35–39.9 (obesity class II)	0.910 (0.644–1.286)	0.6
≥40 (obesity class III)	1.246 (0.653–2.377)	0.5
Diastolic blood pressure		
70–80 mmHg (normal)	1	0.6
<70 mmHg (low)	1.004 (0.808–1.249)	1.0
>80 mmHg (high)	0.891 (0.709–1.119)	0.3
Systolic blood pressure		
120–140 mmHg (normal)	1	0.7
<120 mmHg (low)	1.095 (0.869–1.380)	0.4
>140 mmHg (high)	1.043 (0.852–1.278)	0.7
Albumin/creatinine ratio	1.384 (1.197–1.600)	** <0.001**
Hypertension	0.874 (0.685–1.114)	0.3
Diabetes mellitus	1.669 (1.411–1.976)	** <0.001**
Hyperlipidemia	0.873 (0.736–1.035)	0.1
Tobacco use		**0.004**
Never smokers	1	
Former smokers	1.120 (0.932–1.345)	0.2
Active smokers	1.532 (1.70–2.007)	**0.002**
Alcohol consumption	0.938 (0.782–1.124)	0.5
Physical activity	0.726 (0.580–0.908)	**0.005**
Family history of CAD	0.737 (0.600–0.906)	**0.005**
Prior stroke	1.203 (0.907–1.594)	0.2
Prior MI	1.246 (1.036–1.499)	**0.02**
Previous CABG	1.182 (0.976–1.433)	0.09
Previous PCI	0.920 (0.768–1.101)	0.4
Valvular heart disease	1.823 (1.512–2.198)	** <0.001**
Previous valve replacement	1.033 (0.619–1.721)	0.9
Pacemaker	1.131 (0.877–1.458)	0.3
PAD	1.443 (1.180–1.764)	** <0.001**
Proteinuria	1.147 (0.950–1.385)	0.2
Multivessel coronary artery disease	1.077 (0.872–1.330)	0.5
LVEF		** <0.001**
LVEF, normal (>50%)	1	
LVEF, slightly reduced (41–50%)	1.457 (1.191–1.783)	** <0.001**
LVEF, moderately reduced (31–40%)	1.214 (0.827–1.782	0.3
LVEF, severely reduced (≤ 30%)	2.189 (1.647–2.910)	** <0.001**
PCI performed	0.894 (0.743–1.075)	0.2
CABG performed	1.148 (0.875–1.505)	0.3

BMI, body mass index; CABG, coronary artery bypass graft; CAD, coronary artery disease; CI, confidence interval; CKD, chronic kidney disease; LVEF, left-ventricular ejection fraction; MI, myocardial infarction; PCI, percutaneous coronary intervention; PAD, peripheral artery disease

## Discussion

This analysis of CAD-REF Registry long-term data provides insight into sex-specific disease characteristics, treatment, and mortality of patients with normal and impaired renal function and angiographically proven CAD. Few studies [16, 17] and registries [18] have evaluated patients with CAD and CKD, but sex-specific data and analyses were not reported. Other publications have focused only on sex differences in CAD but lack data on renal function [6, 19, 20]. The inclusion of predominantly male patients, up to 77% as in our registry, is a common feature of these studies and registries. The under-representation of women has long been criticized [21], especially since cardiovascular disease is the number one cause of mortality in both women and men. However, the proportion of women in CAD studies is still at a low, ~ 25%, and thus lower than the female proportion of about 46% in the CAD population [22]. The unequal distribution between men and women in these studies may reflect a lack of awareness of how to identify women eligible for coronary angiography, but it seems to reflect the current reality in this health sector.

### Baseline characteristics, extent of coronary artery disease, and interventions

The German multicenter, prospective, observational CAD-REF Registry confirms the well-known different risk profiles in cardiovascular disease of women in comparison to men: women are usually older, show a higher prevalence of risk factors and comorbidities such as CKD, diabetes mellitus, hypertension, or positive family history of CAD, and had less prior MI and fewer revascularization procedures, higher incidence of preserved LVEF, and lesser extent of CAD (6, 18–20, 23–29). In the CAD-REF Registry, the prevalence of CKD stages 1–2 (51%) or CKD stages 3–5 (30%) in patients with CAD was higher than the prevalence in the general population, e.g., 5.9% prevalence of CKD stages 3–5 in Germany [30]. In particular, CKD stages 3–5 affect women with multivessel disease more frequently than men with multivessel disease. This underlines the urgent need to examine and treat renal comorbidity especially in female patients with CAD.

Conflicting data exit on outcome of women after PCI and CABG. While some researchers found higher in-hospital mortality and worse outcome for women after PCI [31, 32], others reported no sex-specific difference for PCI and CABG [33]. In our registry, in-hospital outcome revealed no sex-specific difference. Many factors contribute to the outcome after interventions: e.g., age, acute or stable CAD, medical treatment, concomitant diseases, experience of the physician, and number of PCIs performed annually at the treatment center. Furthermore, studies, especially randomized-controlled trials, include only highly selected patients, whereas registries include patients from routine clinical practice. All these aspects might explain the different outcomes in diverse registries and studies.

### Drug treatment

Control of blood pressure and atherosclerotic risk factors are the key aspects for cardiovascular disease management, especially in patients with reduced renal function. Our data on drug treatment showed high prescription rates of antihypertensive drugs (> 98%) and acetylsalicylic acid (> 91%) both for women and men after hospital discharge, but insufficient prescription rates of statins which were even lower in females (82%) compared to males (85%). Importantly, our registry showed that prescription patterns according to guidelines [34] are realized to a higher degree than about to 20 years ago [17]. Similarly, the CLARIFY registry which started in 2009 reported better secondary prevention in patients with CKD and CAD with more than 75% of all patients taking ACE inhibitors or angiotensin-receptor blockers, 95% taking antiplatelet medication, and—comparable to our findings—84% taking statins [18]. Consistently, lower treatment with statins in women was reported from researchers of the CLARIFY registry [6] and from the Dyslipidemia International Study (DYSIS) [35].

### Outcome and mortality

The strength of the present analysis is the long-term observation of mortality, since patients are rarely followed up for more than 5 years. During the 8-year follow-up period, we found no difference in mortality between women and men neither overall nor when they were grouped by renal function (no CKD, CKD stages 1–2, CKD stages 3–5). Sex was not an independent predictor for mortality, in contrast to age and comorbidities such as diabetes mellitus, CKD, reduced left-ventricular ejection fraction, valvular heart disease, and peripheral artery disease. Our results show no clear advantage regarding either revascularization technique in CAD patients with CKD in terms of long-term mortality. Not surprisingly, overall-mortality was higher in patients with more advanced CKD than in patients without CKD. Several studies with follow-up periods of 1–5 years support this observation: a very recent international, multicenter registry evaluation on the outcome after contemporary PCI in patients with CAD and renal insufficiency reported that one of the most powerful parameters for adverse outcome, namely major adverse cardiovascular events including cardiac death as well as a patient-oriented composite endpoint including all-cause death, was the presence of CKD and dialysis-dependent CKD [36]. Other predictors were age, diabetes mellitus, previous MI, and smoking, all in good accordance with our findings. The CLARIFY registry [6], evaluating patients with CAD, reported a comparable 1-year outcome for men and women. The 5-year outcome of the same registry also showed no sex-specific difference in all-cause mortality [8]. Similar to our registry, main independent predictors for cardiovascular mortality or non-fatal myocardial infarction were age, diabetes, smoking, prior MI, peripheral artery disease, but also prior stroke, atrial fibrillation, and history of hospitalization for heart failure. A pooled analysis of individual patient data regarding outcome after PCI [37], a subgroup analysis of the GLOBAL LEADERS trial [38], and a sex-related study on patients with acute myocardial infarction [39] also found no association of sex with long-term mortality. In contrast, 10 years ago Ezekowitz et al. [17] reported on a higher 1-year mortality in women with CAD compared to men, but prescription rates for guideline-recommended medication were lower in that study than in our registry. Improved medical treatment strategies might have lowered the mortality risk for women in the last years. Additionally, as mentioned above, other factors such as comorbidities and lifestyle factors have an impact on mortality. Therefore, prevention and treatment of comorbidities such as chronic kidney disease, atherosclerotic disease in general, and diabetes mellitus are essential for lowering mortality. Furthermore, it should be taken into account that personal circumstances might have an influence on outcome: recently, the GENESIS-PRAXY study brought into focus that behavior and characteristics which are traditionally ascribed women influence the outcome of male and female patients with acute coronary syndrome. The researchers showed that young patients with more typical to feminine roles ascribed traits and social roles had worse outcome than patients with a personality traditionally ascribed to men, regardless of their biological sex [40].

### Limitation

In our registry, patients who received a coronary angiogram were consecutively enrolled, resulting in an over-representation of men and an under-representation of women. This unequal sex distribution has been reported earlier for diverse populations undergoing coronary angiography [6, 19, 23, 27, 28] and has been criticized [21]. Therefore, the evidence base for treatment of CAD is more limited for women than for men.

Most characteristics of the patients were only collected at baseline and cardiovascular risk factors were evaluated using questionnaires, not by physical examination. Therefore, the prevalence and incidence of some cardiovascular risk factors, e.g., peripheral artery disease, may be actually higher than recorded. Also, we collected only one serum sample for estimation of GFR. Since serum creatinine concentration depends also on other factors (e.g., muscle mass and nutritional status) than kidney function, some patients may be misclassified. Similarly, proteinuria was detected by dipstick test before angiography which is a semiquantitative estimation of proteinuria and may also lead to misclassification. Blood pressure values were collected with a single measurement at patient hospitalization. The results on the impact of low or high blood pressure levels on mortality are therefore limited.

In long-term outpatient registries, it is difficult to stay in contact with patients over a very long period of time. Therefore, the number of patients for whom follow-up data could be collected decreased over time and cause of death was often unknown. This is the reason why only data on all-cause mortality are presented.

Finally, data were collected in Germany only, an industrialized country with a very sophisticated health care system. The results cannot be extrapolated to other countries or regions with limited medical care.

### Conclusions

Sex differences in patients with CKD and CAD mainly exist in cardiovascular risk profile determined before diagnosis of CAD. Treatment differences between men and women were not observed, which may be the reason for similar in-hospital and long-term outcome. Therefore, sex differences may start to diminish possibly due to the broader use of effective secondary prevention. Nevertheless, further research on sex-specific strategies is warranted to optimize pharmacological and interventional treatment concepts for women and men especially with decreased renal function, since mortality rates in this high-risk group remain high.

## Supplementary Information

Below is the link to the electronic supplementary material.

Supplementary file1 (PDF 191 KB)

## Declarations

### Data availability statement

The data that support the finding of this study are available from the corresponding author upon reasonable request.
