# Supplementary material for: Sex-specific differences and long-term outcome of patients with coronary artery disease and chronic kidney disease: the Coronary Artery Disease and Renal Failure (CAD-REF) Registry
Source: Clin Res Cardiol. 2021 May 26;110(10):1625–36. doi: 10.1007/s00392-021-01864-5 (PMC8484247; doi:10.1007/s00392-021-01864-5)
Supplement: Supplementary file 1 — Supplementary file1 (PDF 191 KB) [file 392_2021_1864_MOESM1_ESM.pdf]

Supplements to

Sex-specific differences and long-term outcome of patients with coronary artery disease and chronic kidney disease: The Coronary Artery Disease and Renal Failure (CAD-REF) Registry

Christiane Engelbertz[a*], PhD; Hans O. Pinnschmidt[b*], PhD; Eva Freisinger[a], MD; Holger Reinecke[a], MD; Boris Schmitz[c], PhD; Manfred Fobker[d], PhD; Roland E. Schmieder[e], MD; Karl Wegscheider[b], PhD; Günter Breithardt[a], MD, EFESC, FACC, FHRS; Hermann Pavenstädt[f], MD; Eva Brand[f], MD, PhD

a Department of Cardiology I – Coronary and Peripheral Vascular Disease, Heart Failure, University Hospital Muenster, Cardiol, Muenster, Germany

b Department of Medical Biometry and Epidemiology, University Medical Centre Hamburg-Eppendorf, Hamburg, Germany

c Institute of Sports Medicine, Molecular Genetics of Cardiovascular Disease, University Hospital Muenster, Muenster, Germany

d Center of Laboratory Medicine, University Hospital Muenster, Muenster, Germany

e Department of Nephrology and Hypertension, University of Erlangen-Nuernberg, Erlangen, Germany

f Department of Nephrology, Hypertension, and Rheumatology, University Hospital Muenster, Muenster, Germany

*contributed equally

Table of Contents

Definition of cardiovascular risk factors

Supplementary Table 1

Supplementary Table 2

Supplementary Figure 1

Definition of cardiovascular risk factors

Arterial hypertension was documented by questionnaire. Hyperlipidemia was defined as a total plasma cholesterol >5.2 mmol/l (>200 mg/dl), plasma low-density lipoprotein cholesterol >3.87 mmol/l (>150 mg/dl), or plasma lipoprotein (a) >250 mg/l; diabetes mellitus definition was based on oral antidiabetic medication or insulin treatment; physical inactivity was defined by no, irregular, or seldom physical activity for less than 30 minutes per week. Alcohol consumption was documented by questionnaire and was defined as occasional, regular, or excessive alcohol consumption. Peripheral artery disease was defined according to the Fontaine classification (stages I, IIa, IIb, III, and IV). Family history of CAD was assumed if parents, siblings, or children had previous myocardial infarction, stroke, coronary artery bypass grafting (CABG), or stent implantation.

Supplementary Table 1: Patient characteristics and renal laboratory parameters at the time of enrolment (age-adjusted analysis)

	Overall population	Women	Men	$p_{age\text{-}adjusted}$
**Baseline parameters**				
Patients, n (% of all)	3,352 (100)	773 (23)	2,579 (77)	
Age, mean (95% CI), years<br>n (women) 773, n (men) 2,579	67 (67-67)	70 (69-71)	66 (66-67)	**<0.001**[a]
Age ≤50 years, n (%)	258 (7.7)	42 (5.4)	216 (8.4)	**0.008**[a]
BMI, mean (95% CI), kg/m^2<br>n (women) 767, n (men) 2,567	28.3 (28.1-28.4)	28.4 (28.1-28.8)	28.2 (28.1-28.4)	0.05
WHR, mean (95% CI)<br>n (women) 540, n (men) 1,772	0.99 (0.98-0.99)	0.94 (0.93-0.95)	1.00 (1.00-1.00)	**<0.001**
Systolic blood pressure, mean (95% CI), mmHg<br>n (women) 772, n (men) 2,574	134 (134-135)	136 (134-137)	134 (133-135)	0.07
Diastolic blood pressure, mean (95% CI), mmHg<br>n (women) 772, n (men) 2,572	77 (76-77)	76 (75-77)	77 (76-77)	0.42
Pulse pressure, mean (95% CI), mmHg<br>n (women) 772, n (men) 2,572	58 (57-58)	60 (59-61)	57 (56-58)	**0.005**
**Cardiovascular risk factors**				
Arterial hypertension, n (%)	2,794 (83)	676 (87)	2,118 (82)	**0.04**
Diabetes mellitus, n (%)	856 (25)	223 (29)	633 (25)	0.15
Hyperlipidemia, n (%)	2,178 (68)	500 (68)	1,678 (68)	0.52
Tobacco use, n (%)	1,769 (54)	242 (32)	1,527 (61)	**<0.001**
Alcohol consumption, n (%)	1,611 (59)	272 (43)	1,339 (64)	**<0.001**
Physical inactivity, n (%)	2,078 (76)	498 (80)	1,580 (75)	0.08
Family history of CAD, n (%)	1,176 (43)	292 (46)	884 (42)	**0.001**

**Cardiovascular events**				
Previous stroke, n (%)	188 (6)	43 (6)	145 (6)	0.43
Previous MI, n (%)	1,086 (32)	205 (27)	881 (34)	**<0.001**[a]
Previous CABG, n (%)	682 (20)	126 (16)	556 (22)	**<0.001**
Previous PCI, n (%)	1,494 (45)	308 (40)	1,186 (46)	**0.001**
Valvular heart disease, n (%)	454 (14)	125 (16)	329 (13)	0.77
Previous valve replacement, n (%)	48 (1.4)	11 (1.4)	37 (1.4)	0.63
Pacemaker, n (%)	232 (7)	49 (6)	183 (7)	0.05
PAD, n (%)	350 (10)	78 (10)	272 (11)	0.12
**Renal laboratory parameters**				
Creatinine, mean (95% CI), mg/dl<br>n (women) 773, n (men) 2,579	1.1 (1.0-1.1)	0.9 (0.9-1.0)	1.1 (1.1-1.1)	**<0.001**
eGFR, mean (95% CI), ml/min/1.73 m^2<br>n (women) 773, n (men) 2,579	72 (71-72)	66 (64-67)	73 (72-74)	**<0.001**
Proteinuria, n (%)	637 (19)	151 (20)	486 (19)	0.98
Albumin/creatinine-ratio, mean (95% CI), mg/g<br>n (women) 349, n (men) 1,199	34 (32-36)	40 (35-47)	32 (30-35)	0.12
Protein/creatinine-ratio, mean (95% CI), mg/g<br>n (women) 721, n (men) 2,392	130 (127-134)	170 (160-181)	120 (117-124)	**<0.001**

For all variables that are age dependent ($p_{age}<0.05$), the p-value after age-adjusted analysis $p_{age\text{-}adjusted}$ is given. For all variables that are not age dependent ($p_{age}\geq 0.05$), the unadjusted p-value is given.

[a] denotes unadjusted p-value

BMI, body mass index; CABG, coronary artery bypass graft; CAD, coronary artery disease; CI, confidence interval; eGFR, estimated glomerular filtration rate determined by CKD-EPI formula; MI, myocardial infarction; PCI, percutaneous coronary intervention; PAD, peripheral artery disease; $p_{age\text{-}adjusted}$: p-value after age-adjusted analysis; WHR, waist-to-hip ratio.

Supplementary Table 2: Cardiological data, treatment after/during index angiography, in-hospital complications and discharge (age-adjusted analysis)

	Overall population	Women	Men	$p_{age-adjusted}$
Patients, n (% of all)	3,352 (100)	773 (23)	2,579 (77)	
**Cardiological data**				
Indication for coronary angiography, emergency intervention, n (%)	714 (21)	167 (22)	547 (21)	0.24
Multivessel coronary artery disease, n (%)	2,494 (74)	522 (68)	1,972 (76)	**<0.001**
LVEF, n (%)	2,727			**<0.001**
Normal (>50%)	1,712 (63)	450 (73)	1,262 (60)	
Slightly reduced (41-50%)	621 (23)	106 (17)	515 (24)	
Moderately reduced (31-40%)	169 (6)	38 (6)	131 (6)	
Severely reduced (≤30%)	225 (8)	26 (4)	199 (9)	
**Treatment after/during index angiography**				
PCI performed, n (%)	2,281 (68)	538 (70)	1,743 (68)	**0.04**
Performed stenting, n (%)	1,984 (87)	472 (88)	1,512 (87)	0.65
Intervened arteries (LAD, LCX, RCA)				0.52[a]
One, n (%)	1,788 (90)	423 (90)	1,365 (90)	
Two, n (%)	177 (9)	43 (9)	134 (9)	
Three, n (%)	19 (1.0)	6 (1.3)	13 (0.9)	
CABG performed, n (%)	344 (10)	80 (10)	264 (10)	0.60

**In-hospital complications**				
PCI after index intervention, n (%)	124 (4)	23 (3)	101 (4)	0.35
CABG after index intervention, n (%)	98 (3)	20 (3)	78 (3)	0.32
Requiring dialysis after index intervention, n (%)	5 (0.1)	1 (0.1)	4 (0.2)	0.87[a]
MI during/after index intervention, n (%)	7 (0.2)	2 (0.2)	5 (0.3)	0.73[a]
Stroke after index intervention, n (%)	5 (0.1)	2 (0.3)	3 (0.1)	0.38[a]
**Outcome**	3,350	773	2577	0.34
Discharged alive, n (%)	3,037 (91)	697 (90)	2,340 (91)	
In-hospital death, n (%)	8 (0.2)	4 (0.5)	4 (0.2)	
Discharged to another hospital/medical rehabilitation measures, n (%)	305 (9)	72 (9)	233 (9)	

Multivessel coronary artery disease covers two-, three-vessel disease and main stem disease.

For all variables that are age dependent (p_{age}<0.05), the p-value after age-adjusted analysis $p_{age\text{-}adjusted}$ is given. For all variables that are not age dependent (p_{age}≥0.05), the unadjusted p-value is given.

[a] denotes unadjusted p-value

CABG, coronary artery bypass graft; LAD, left anterior descending artery; LCX, left circumflex artery; LEVF, left ventricular ejection fraction; PCI, percutaneous coronary intervention, $p_{age\text{-}adjusted}$: age-adjusted p-value; RCA, right coronary artery.

Supplemental Figure 1

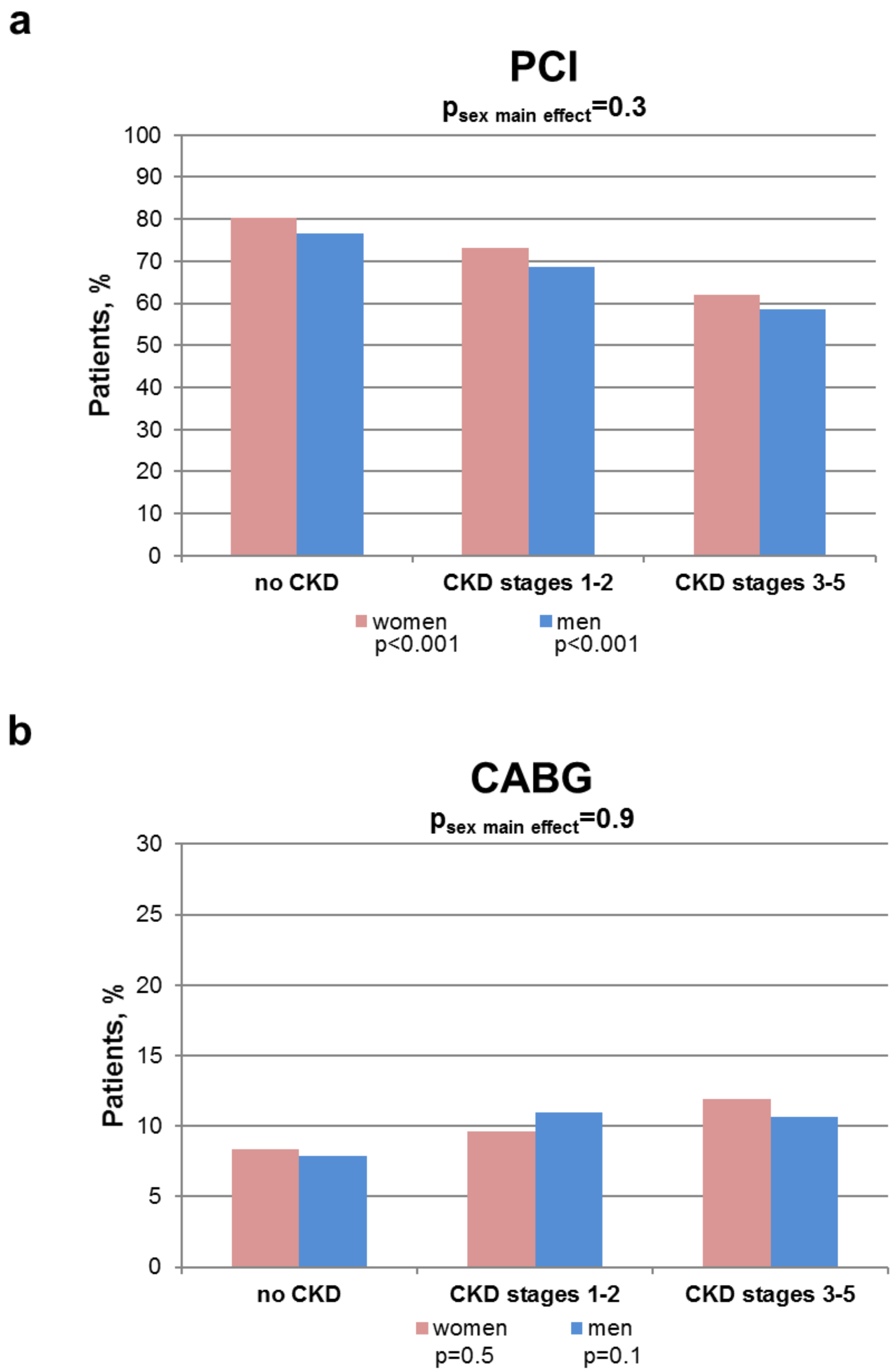

a) Female and male patients with percutaneous coronary intervention (PCI) after angiography according to renal function. There was no significant difference between women and men ($p_{sex\ main\ effect}$=0.3). Separate analysis of women and men showed a significant difference of performed PCI in women and in men (both p<0.001, chi square test) according to CKD severity.

b) Female and male patients with coronary artery bypass graft (CABG) after angiography according to renal function. There was no significant difference of performed CABG between men and women ($p_{sex\ main\ effect}$=0.9), nor in separate analysis of women or of men according to renal function (p=0.5 for women and p=0.1 for men, chi square test).
